# Evaluating Gait Impairment in Parkinson’s Disease from Instrumented Insole and IMU Sensor Data

**DOI:** 10.3390/s23083902

**Published:** 2023-04-12

**Authors:** Vassilis Tsakanikas, Adamantios Ntanis, George Rigas, Christos Androutsos, Dimitrios Boucharas, Nikolaos Tachos, Vasileios Skaramagkas, Chariklia Chatzaki, Zinovia Kefalopoulou, Manolis Tsiknakis, Dimitrios Fotiadis

**Affiliations:** 1 Unit of Medical Technology and Intelligent Information Systems, Department of Materials Science and Engineering, University of Ioannina, GR 45110 Ioannina, Greece; 2 PD Neurotechnology Ltd., GR 45500 Ioannina, Greece; 3 Biomedical Research Institute, Foundation for Research and Technology—Hellas, GR 45500 Ioannina, Greece; 4 Institute of Computer Science, Foundation for Research and Technology—Hellas, GR 70013 Heraklion, Greece; 5 Department of Electrical and Computer Engineering, Hellenic Mediterranean University, GR 71004 Heraklion, Greece; 6 Department of Neurology, General University Hospital of Patras, GR 26504 Patras, Greece

**Keywords:** Parkinson’s disease, gait analysis, instrumented insoles, IMU sensors, digital biomarkers, sensor fusion, wearable sensors

## Abstract

Parkinson’s disease (PD) is characterized by a variety of motor and non-motor symptoms, some of them pertaining to gait and balance. The use of sensors for the monitoring of patients’ mobility and the extraction of gait parameters, has emerged as an objective method for assessing the efficacy of their treatment and the progression of the disease. To that end, two popular solutions are pressure insoles and body-worn IMU-based devices, which have been used for precise, continuous, remote, and passive gait assessment. In this work, insole and IMU-based solutions were evaluated for assessing gait impairment, and were subsequently compared, producing evidence to support the use of instrumentation in everyday clinical practice. The evaluation was conducted using two datasets, generated during a clinical study, in which patients with PD wore, simultaneously, a pair of instrumented insoles and a set of wearable IMU-based devices. The data from the study were used to extract and compare gait features, independently, from the two aforementioned systems. Subsequently, subsets comprised of the extracted features, were used by machine learning algorithms for gait impairment assessment. The results indicated that insole gait kinematic features were highly correlated with those extracted from IMU-based devices. Moreover, both had the capacity to train accurate machine learning models for the detection of PD gait impairment.

## 1. Introduction

Gait is a basic characteristic of human life deeply tied to the quality of life [1]. While it is usually taken for granted, gait as an activity is complex and rich in information. The analysis of the human gait is the sequential analysis of movement, and it consists of the estimation and assessment of the quantified characteristics of human locomotion. In a clinical context, information regarding an individual’s gait, is paramount to diagnosing various underlying medical conditions, especially neurological, further enabling the monitoring of the progression of the symptoms, as well as the overall quality of life of patients [2]. More specifically, in the case of PD, evaluating the extent of an individual’s gait impairment is one of the most important aspects of the neurological examination [3]. At the same time, it is also the most daunting. Gait disturbances exhibited in Parkinson’s disease are multifaceted, with their manifestations being hard to identify, as they change and increase in complexity, depending on the disease’s stage [1,3].

The current standard practice for mobility evaluation in Parkinson’s disease is based on in-person, observational, clinical assessments with the help of validated, standardized questionnaires (such as the Unified Parkinson’s Disease Rating Scale— UPDRS) [4,5]. As physicians are evaluating mobility based on their experience, this procedure is inaccurate, and to a large extent subjective [2]. Moreover, the evaluation itself, normally performed in a hospital, clinic, or physician’s office, cannot capture the full spectrum of a patient’s symptomatology. Certainly, it cannot provide a holistic view regarding the magnitude of the symptoms during the day, when the patients perform their daily activities in their environment. Clinical mobility evaluation has not progressed enough through the years because the most important factors physicians take into consideration are the time it takes to evaluate the mobility of a patient [4], as well as the complexity of the equipment they use [2].

Ideally, mobility evaluation for Parkinson’s disease should be instrumented and based on a continuous monitoring paradigm, that follows patients throughout their day, capturing rich information in their natural environment. Monitoring patients during their normal daily routines is essential for understanding their state, as well as the progression of their symptoms, especially in diseases with complex symptomatology and fluctuating motor symptoms [6]. Gait analysis in research settings has come a long way, facilitated in part by advancements in affordable and easy-to-use instrumentation [2,5], as well as new methods for gait analysis. Some examples include methods for IMU sensors [7,8], pressure insoles [9], radars [10], cameras [11,12], etc. Through instrumentation, a plethora of gait parameters has been identified (e.g., spatiotemporal, kinematic, kinetic, etc.) [4,5,13] enabling the quantification of human gait of a diverse set of populations (e.g., healthy or patient individuals, etc.) [4,14].

It is evident that there is a disparity in the quality of information acquired through gait analysis between clinical and research contexts, hence there is a need for clinicians to move forward and update their practices. Currently, two of the best approaches towards this endeavor lie in the development and use of medical devices for clinical gait analysis using instrumented insoles and IMU sensors. IMU sensors have recently been integrated into clinical practice as the most frequently used type of sensor for gait analysis [15] and, maybe more importantly, into the homes of patient populations, enabling reliable, real-time, continuous monitoring [16]. On the other hand, pressure insoles are the second most frequently used sensor for monitoring gait, although they are most often used as the baseline for validating data from IMU sensors [15]. In any case, both types of devices (insoles and IMU-based) are currently dominating the landscape of clinical gait analysis, albeit, usually complimenting one another (i.e., insoles acting as the ground truth for the IMU sensors), or being utilized in tandem as building blocks of various devices. The latter approach provides even richer motion data by combining the strength of both sensors [15].

Data from instrumented insoles and IMU sensors have been utilized in many applications for monitoring the mobility of impaired patients with Parkinson’s disease. Signal processing and statistical techniques have been used, with data generated from instrumented insoles, for the evaluation of gait impairment symptoms exhibited in Parkinson’s disease, as well as for quantifying weight-bearing, balance, and mobility of patients, in both everyday scenarios and standardizing test setups (TUG test) [17,18]. Similarly, IMU-sensor data have been used for extracting gait events and spatiotemporal gait characteristics, such as initial foot contact (IC) and final foot contact (FC) in patients with Parkinson’s disease [19,20,21]. Machine learning techniques have been implemented for the classification of tasks such as differentiating between healthy subjects and patients, as well as for motor status discrimination [5,22,23]. All in all, IMU sensors, alongside insole solutions, have enabled the development of medical devices [16,24], that are not only accurate and affordable, but also easily and comfortably wearable.

Having seen the strides made recently by the research community in gait analysis, along with the obstacles impeding their adoption by physicians during routine clinical practice, there is a realization that there is a significant need for further evidence. This work tries to fill this gap by evaluating both instrumented insoles and wearable IMU-based monitoring devices for assessing gait impairment in patients with Parkinson’s disease. Specifically, the aim of this endeavor is to provide new insights by achieving the following goals: First, to compare the performance of instrumented pressure insoles and IMU sensors when applied to the same task. The accuracy and robustness of their outputs will be evaluated separately. Second, to combine motion data acquired from instrumented pressure insoles and IMU-based monitoring devices with the goal of providing a more comprehensive assessment of gait characteristics in impaired patients. This sensor fusion approach will augment the extracted gait features and offer physicians a highly detailed representation of the patient’s motor symptoms. Third, to evaluate the strengths and weaknesses of each system considering specific clinical use cases.

## 2. Materials and Methods

The evaluation of the IMU and the insole data for gait assessment of patients with Parkinson’s disease was conducted using a dataset generated during a study employing the “Smart-Insole Gait Assessment Protocol” [25]. The primary goal of the protocol was the evaluation of motor symptoms exhibited in the lower extremities of patients with Parkinson’s disease. The parts of the protocol that are relevant to this work, as well as the participants that took part in the study, are described in Section 2.1. The insole- and the IMU-generated data, along with the data processing pipelines, resulting in extracted gait features are explained in Section 2.2 and Section 2.3 respectively. The methods used for gait impairment detection are presented in Section 2.5.

### 2.1. Test Protocol and Participants

For the study, 19 patients diagnosed with Parkinson’s disease were recruited and subsequently requested to complete a set of 5 specialized tests, while wearing both a pair of pressure insoles and a set of wearable IMU sensors. Each patient performed the tests in both the ON and the OFF states. In all cases, the participants were recorded on video and observed by neurologists, specializing in movement disorders. The neurologists evaluated the performance of each participant based on the MDS-UPDRS questionnaire [26]. The video recordings were used solely for the evaluation of the participants, as well as for annotating the dataset. Information regarding the demographics of the participants, their disease status, and medication is presented in Table 1. For the purposes of this work, data generated during the “Walk Straight and Turn Test” (described in detail in [25]) were used, along with expert ratings of the Item 3.10 (Gait) of the MDS-UPDRS questionnaire. During the test, the participants had to walk in a straight line for 10 m, then turn around and return to their original starting position. The test was repeated twice (per patient state) and performed at three different speeds (slow, normal, and fast). The distribution of the expected ratings of Item 3.10 (Gait) for the 19 patients is presented in Table 2.

### 2.2. Insole Data and Processing

**Figure 1:**
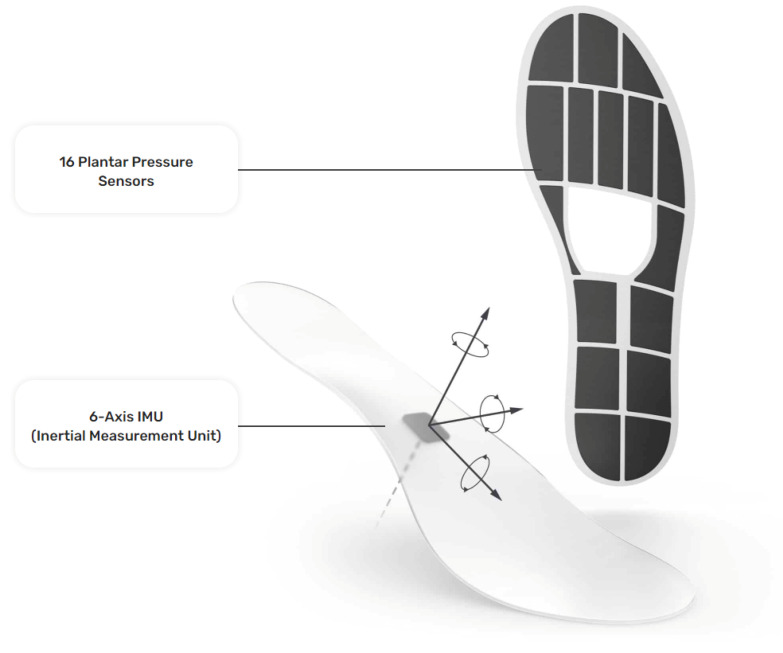
The instrumented insole (Moticon ReGo Model Insole 3) is used to generate the data for the Smart-Insole dataset. Reprinted with permission from [28] Moticon ReGo AG.

The insole data were generated with a pair of Moticon ReGo instrumented insoles (specifically, Model Insole 3), manufactured by Moticon ReGo AG (Munich, Germany). The insoles were fitted inside a pair of off-the-shelf lightweight and flexible shoes. Figure 1 presents the position of the pressure and the IMU sensors in an insole, with each black cell in the image representing a pressure sensor. Those insoles included 16 capacitive-type pressure sensors, as well as a 6-axis Inertial Measurement Unit (IMU) sensor capturing  acceleration and angular velocity information. All the data derived from the pressure insoles were calibrated at the initiation phase of the data collection by using a zeroing function provided by Moticon ReGo AG. The data recording was performed with a sampling rate of 100Hz and each recording included a total of 51 features. From those 51 features, 25 correspond to features extracted from the left insole, and 25 from the right insole, with the remaining feature being the timestamp of the data acquisition. Each instrumented insole generated the following data: a pressure value for each of the 16 pressure sensors (N/cm^2^), an acceleration (g) and angular velocity (dps) value for each of the x, y, and z axes, the computed center of pressure in the x, y coordinates. The center of pressure was derived by a proprietary, validated, algorithm of Moticon ReGo AG. Moreover, it ranges from −0.5 to 0.5 and it is related to the insole’s length/width, as well as the total force computed by the instrumented insole (N) [27].

The raw signals generated from the instrumented insoles were used to extract gait features based on the model described in [17]. The model included a processing pipeline that is briefly explained below. First, the raw signals went through a pre-processing step, that implemented normalization and noise removal. Next, the main processing phase was initiated, calculating for each gait cycle, the time duration between successive gait events. The gait events in question can be seen in Table 3 and were identified based on a set of conditions presented in [27] (also seen in Table 3). Finally, using the timing of the gait events, the model produced, for each gait cycle, the set of gait features, listed in Table 4.

### 2.3. IMU Data and Processing

**Figure 2:**
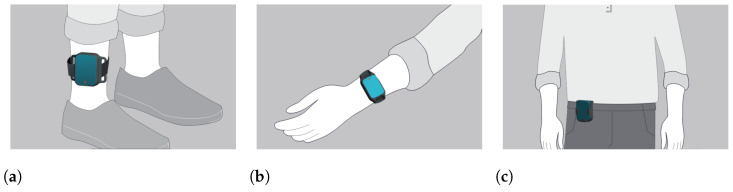
Placement of the wearable IMU-based monitoring devices. (**a**): Placement of ankle sensors. (**b**): Placement of wrist sensors. (**c**): Placement of the waist sensor.

The IMU-sensor data were generated by a set of 5 wearable sensors which are part of the PDMonitor^®^ medical device, manufactured by PD Neurotechnology Ltd. (London, UK). The device includes five lightweight wearable sensors, or MDs (Monitoring Devices), used to collect raw kinematic data. Each MD is 41 mm × 30.6 mm × 12.85 mm and is based on a 9-degree IMU sensor acquiring data with a sampling frequency of 59.5Hz. Two of the monitoring devices are placed on the wrists, two on the ankles, and one on the waist (Figure 2). The rest of the system components are described in detail in [24]. The intended use of the PDMonitor^®^ system is to monitor the symptoms of patients with Parkinson’s disease but for the purposes of the conducted tests, the PDMonitor^®^ system was not used as intended but only for logging motion data and extracting gait features.

Each recording, with one of the 5 sensors, of the PDMonitor^®^ includes data corresponding to values of 9 signals, along with a timestamp denoting the time each measurement was conducted. In summary, the generated data are the following: a Unix timestamp logging the time of data acquisition, along with an acceleration (g), angular velocity (dps), and geomagnetic field (T) value for each of the x, y, and z axes.

From the dataset, only data captured from the 2 ankle IMU-based monitoring devices were used for the purposes of this work. The gait features of interest did not require data from either the wrist or the waist sensors. For gait analysis of healthy individuals, in general, one ankle sensor would be enough, as their gait characteristics would be similar for both feet. But, in this case, the wearers included patients with gait impairment, thus monitoring both of their legs, individually, while walking was necessary. Moreover, a second ankle sensor enabled the identification of the first and the last step of each participant during the 2 conducted testing scenarios.

**Figure 3:**
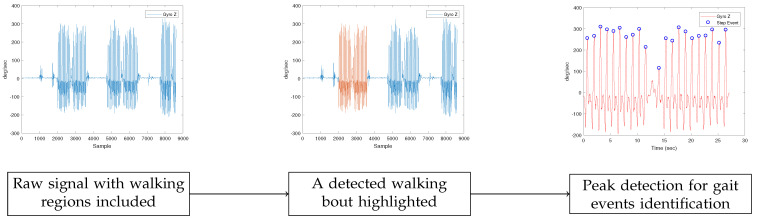
IMU-based gait region identification. In the first plot, the initial signal is shown. For the detection of gait events in the specific dataset, the gyroscope’s Z-axis signal was employed. Next, in the second plot, the result of the procedure for the detection of walking bouts appears, with the region of interest highlighted. In our work, the regions were extracted manually. Finally, in the third plot, the results of the peak detection method can be seen. The peak detection method was used to identify gait events.

The gait features were extracted from the IMU-generated dataset using a processing pipeline comprised of 3 main steps. The first step was the identification of the walking bouts for each participant through the gyroscope data, as can be seen in the second plot of Figure 3. The walking bouts on the recorded signals were annotated manually based on the time information of each test. It should be noted that gait detection from IMU data could be also performed automatically.

Next, gait events were detected after applying peak detection in the readings of the Z axis of each of the ankles’ gyroscopes (using the MATLAB’s findPeaks method), presented in the third plot of Figure 3).  Those gait events were the maximum swing (MS), the toe-off (TO), and the initial contact (IC). The MATLAB’s findpeaks function was used with three different threshold levels (parameter MaxPeakHeight), corresponding to three different gait cycle events:

- The maximum swing (MS), found through peak detection based on the Z axis signal of the gyroscope ($s_{z}$), with a minimum peak distance of 0.5 s and a minimum peak width of 0.1 s:
  (1) $$t_{\mathrm{MS}}=\max\left[50,0.3\;\cdot \;\max(s_{z})\right]$$
- The toe-off (TO), identified using peak detection based on the inverted Z-axis signal of the gyroscope ($s_{z}$), with a fixed threshold, as follows:
  (2) $$t_{\mathrm{TO}}=15$$
- The initial contact (IC), found through peak detection based on the inverted Z-axis signal of the gyroscope ($s_{z}$), again with a fixed threshold, as can be seen below:
  (3) $$t_{\mathrm{IC}}=-5$$

**Figure 4:**
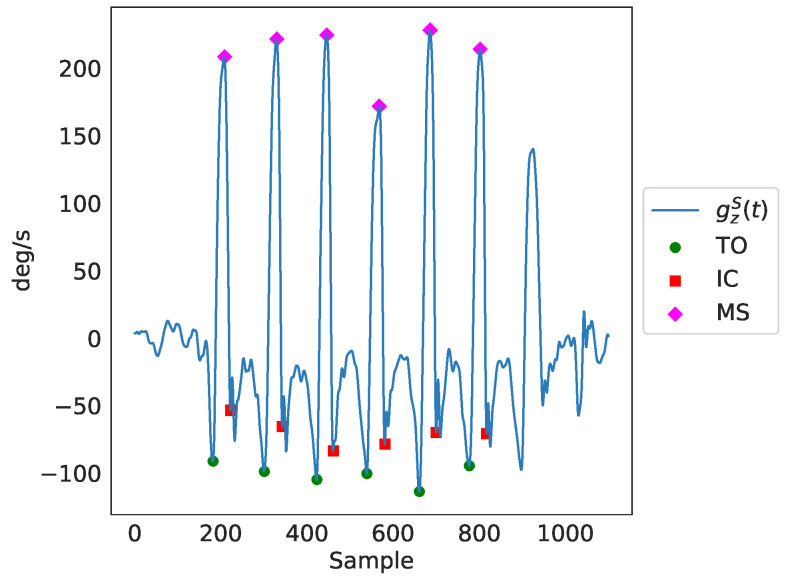
A detailed presentation of the basic gait cycle events, identified through peak detection in the readings of the Z axis of the ankle gyroscopes denoted as $g_{z}^{s}\left(t\right)$ in the figure. TO is the toe-off event, IC is the initial contact event and MS is the maximum swing event.

The identified peaks as a result of the peak detection process can be seen in Figure 4. Finally, through the timing of the gait events, ($T^{\mathrm{MS}}$ for maximum swing, $T^{\mathrm{TO}}$ for toe-off and $T^{\mathrm{IC}}$ for initial contact), as well as through further utilization of the gyroscopes’ and accelerometers’ data, temporal gait features were extracted. In total, there were 141 features extracted, including several spatiotemporal ones. It should be noted that we only employed features that can be estimated from a single IMU sensor (i.e., right/left side stance phase, single support, etc.), since this is a common practice in ambulatory settings. As a result, features that require the use of both IMU sensors (for example, double support) were not included in the feature set. Nevertheless, for each feature, the average value of both legs was included in the feature set. For the comparison analysis of features generated from data of both sources presented in Section 3.1, the features that were extracted from data of IMU-based devices were averaged over both legs.

### 2.4. Comparison of Extracted Features

The set of features that were extracted from the data of the IMU-based devices was compared with the set of features that were extracted from the data of the pressure insoles. As a pre-processing step, the features calculated from the data of the IMU-based monitoring devices were scaled to the range of the corresponding features, calculated from the instrumented insoles. Next, a number of cases in the dataset, from both systems, with no, or very few steps (less than 3), were excluded from the subsequent analysis. As a result, there were a total of 95 cases (test sessions) included for further analysis. Finally, a number of 4 outliers (4/95 or <5%) with “abnormal” values were identified. Those outliers were excluded from the calculated correlation coefficients.

The first analysis performed, concerning the collected data from both described systems (insoles and IMU-based devices), included the Pearson correlation coefficient between the common features extracted from data of both sources. The second analysis, complementary to the calculation of the correlation values, included the assessment of the inter-rater reliability. For this purpose, two methods were used. Initially, the intra-class correlation coefficient (ICC) for single, fixed raters was calculated, assuming as separate data classes the three different speeds (slow, normal, and fast) patients were instructed to perform the relative tests with. Next, a Bland-Altman analysis was conducted to evaluate the agreement between the two different systems, regarding the features extracted from the data generated from them.

### 2.5. ML Methods for Gait Impairment Assessment

In addition to the statistical comparison of the gait features derived by the two sensing systems, described in Section 2.4, a study has been performed, aiming to identify the capacity of these features to train machine learning (ML) models for accurately predicting gait impairment, in terms of MDS-UPDRS item 3.10 result estimation. More specifically, features extracted from the data of the pressure insoles and the IMU sensors were used to differentiate between patients with no gait impairment and patients that exhibited gait impairment (MDS-UPDRS Item 3.10 > 0). For this analysis, only the “Normal” speed data of the participants of the “Walk Straight and Turn Test” iterations were employed along with the expert MDS-UPDRS Item 3.10 ratings. For this binary classification task, the following algorithms were assessed and subsequently compared: Support Vector Machine, Random Forest, Gradient Boosting, and AdaBoost. Three sub-analyses were conducted, each one evaluating those algorithms using a different set of features. Those were:

- features extracted solely from insole data,
- features extracted solely from IMU data,
- features extracted from data of both systems.

The scope of the latter analysis was to identify the maximum accuracy ML models could achieve for correctly assessing gait impairment. Such an ML model’s performance can act as the “baseline” for identifying the loss of accuracy when using data from the two sources separately.

## 3. Results

The following sections present the results of the analyses performed on the collected data. First, the results regarding the comparison of the extracted features are presented, with those of the gait impairment detection analysis following afterward.

### 3.1. Comparison of Extracted Features

**Figure 5:**
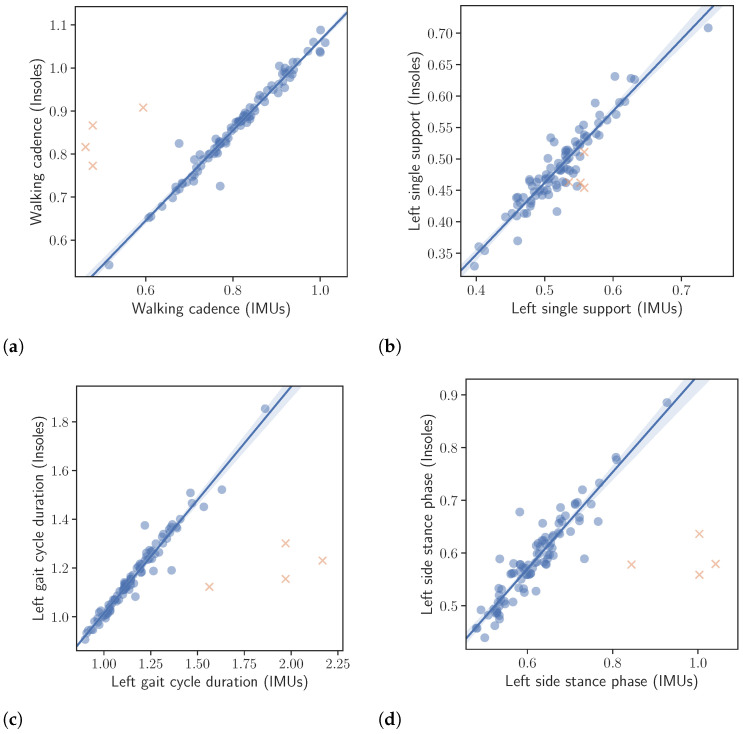
Correlation between the extracted gait kinematic features from the data of the instrumented insoles and the IMU-based devices. Note that the orange crosses represent outliers. The outliers were not taken into account in the calculation of the regression line or the correlation values presented in the text. (**a**): Walking cadence. (**b**): Left single support. (**c**): Left gait cycle duration. (**d**): Left side stance phase.

The results of the correlation analysis described in Section 2.4 are presented in Table 5, while correlation plots, corresponding to the left foot, are depicted in Figure 5. All correlation results were excellent with only the right single support, being slightly lower. More specifically, the calculated correlations were as follows. Left and right gait cycle duration r=0.98 and r=0.97, left and right single support r=0.94 and r=0.86, left and right stance phase r=0.93 and r=0.88, walking cadence r=0.98 and finally number of steps r=0.94.

**Figure 6:**
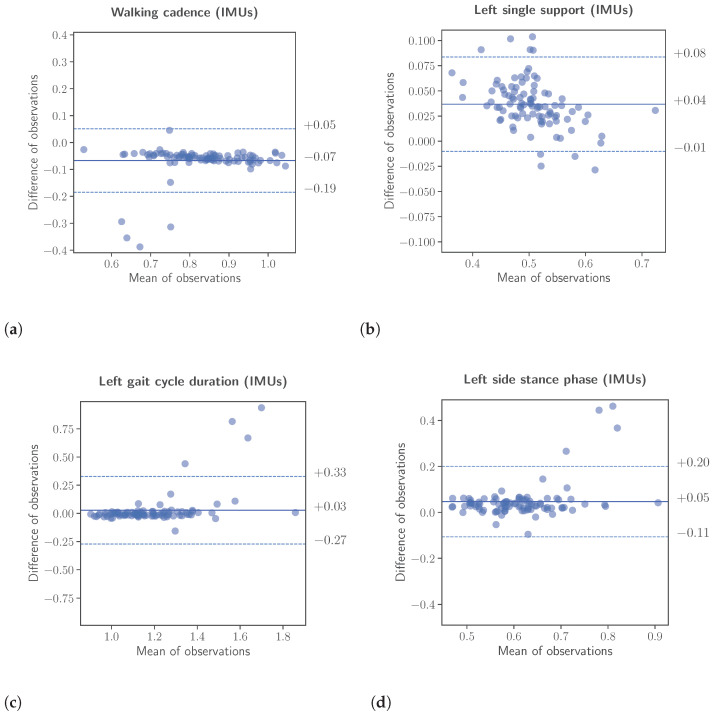
Bland-Altman analyses between the extracted gait kinematic features from the data of the instrumented insoles and the IMU-based devices. The upper and lower limits of the Bland-Altman analyses were calculated using the formula ±1.96·SD, respectively. (**a**): Walking cadence. (**b**): Left single support. (**c**): Left gait cycle duration. (**d**): Left side stance phase.

Complimentary to the correlation analysis, an assessment was conducted to evaluate the inter-rater reliability. The assessment was performed using two different methods, namely the calculation of the intraclass correlation coefficient (ICC) for single, fixed raters and the Bland-Altman analysis. Again for the inter-rater reliability, the features calculated from the data of the IMU-based system were scaled to the range of the corresponding features, calculated from the insole system. The results of the inter-rater reliability analysis are presented in Table 5 as well, while Bland-Altman plots are depicted in Figure 6. For the case of the intraclass correlation coefficients, the ICC values were calculated, as already mentioned, assuming as separate data classes the three different speeds (slow, normal, and fast) patients were instructed to perform the “Walk Straight and Turn Test” with. The results listed in the aforementioned table are as follows. Left and right gait cycle duration ICC=0.98 and ICC=0.96, left and right single support ICC=0.91 and ICC=0.83, left and right stance phase ICC=0.92 and ICC=0.91, walking cadence ICC=0.97 and finally number of steps ICC=0.91. According to [29], ICC values greater than 0.9 are considered excellent reliability, and values greater than 0.75 as good reliability.

### 3.2. ML Methods for Gait Impairment Assessment

As discussed in Section 2.5, for this analysis only the “Normal” speed data of the participants of the “Walk Straight and Turn Test” iterations were utilized. The results for the three feature groups (Insoles Only, IMUs Only, Insoles and IMUs) are presented in Table 6 listing for each classifier the AUC, Accuracy, F1-Score, Precision, and Recall metrics. The results indicate that all algorithms exhibited excellent performance differentiating between healthy and gait-impaired individuals, no matter the feature set they used or the performance metric is chosen in evaluating them. Nonetheless, the features extracted from the data of the pressure insoles, as well as the combination of features extracted from both devices’ data, resulted in a slightly better performance of all algorithms than the features extracted solely from the IMU data.

More specifically, when considering the AUC as the primary metric for the evaluation, the SVM model outperformed the rest of the models in all three analyses. Its performance in the analysis utilizing features extracted from data of both IMU and pressure insoles was 93%, while dropping by 1% and 8% when omitting pressure-related and IMU-related features, respectively. The performance reduction is lower when considering the accuracy (≈4%) or the F1-score (≈4%), indicating that both the IMU-based and the pressure-based features present similar capacity for training machine learning models for the classification between healthy and PD patients.

Along with the aforementioned analysis, a machine learning explainability framework, the Shapley Additive Explanations (SHAP) [30], has been utilized to assess the importance of each feature to the output (prediction) of the aforementioned classifiers. The results are presented in Table 7. Based on Table 7, when utilizing features from both systems, 5 out of 10 most important features were derived from the IMU data. Thus, despite the fact that instrumented insoles are commonly considered the gold standard for gait monitoring, the discriminating power of the IMU-based system was showcased for classifying healthy and impaired individuals, in terms of gait disorders manifested due to Parkinson’s disease. Additionally, based on the relative SHAP values, loading response times appear to play an important role in the machine learning models derived from features extracted from data of both IMU-based and insole systems. The results indicate that gait quality degradation due to Parkinson’s disease mainly involves changes in both the left and right loading response times, thus in the pre-swing times for both feet as well.

## 4. Discussion

Neurological disorders involving movement impairment, like Parkinson’s, have a major effect on the quality of life of patients. Objective measurement applied to movement disorders, especially when pertaining to monitoring gait, can result in physicians better assessing the symptoms of patients and subsequently mitigating them or, more generally, in better managing their condition. Sensing devices, mainly those enabling remote, continuous monitoring, can assist patients and physicians through the frequent collection of movement data. These data can feed machine learning models which can, in an automated fashion, assess the condition of patients based on specific endpoints, such as gait impairment, and raise warnings upon the identification of any possible abnormal condition. Sensing systems can utilize various types of sensing devices, such as the pressure insoles and the IMU sensors used in this work.

When it comes to gait assessment, pressure insole systems can deliver, quality data which can result in the extraction of a large set of gait-related features. Such systems provide the flexibility to collect data from repeated foot strikes in any environment without restrictions placed by the setting. Plantar loading parameters obtained from insole pressure sensors have shown high reliability across multiple trials of the same individual, with low variability between steps [31], and high repeatability between testing days [32]. Analysis of ground reaction force parameters indicated that these measures are also reliable over a range of walking speeds and stride frequencies when collected using pressure sensors [33]. However, a comparison of the two most popular insole pressure measurement systems shows that the accuracy and precision of these systems may be sensitive to the levels of applied pressure, calibration procedure, duration of pressure application, as well as to the insole’s age, and a result its deterioration [34]. Although insole systems have many advantages, they are accompanied by a few significant drawbacks. First of all, insoles lead to a poor user experience and satisfaction, due to the low battery life and raised temperature levels on the surface making contact with the wearers’ feet. As a result, systems based on pressure insoles are not usually recommended for home-based environments, but rather for clinical settings, in which case their use is limited to a given scenario and time duration. Another drawback, adding to the poor user experience of insoles, is that patients, frequently, feel uncomfortable when they fit them in their footwear. On top of that, pressure insoles must be specifically selected for each individual patient, according to their foot size, thus reducing their re-usability in general.

On the other hand, inertial measurement unit (IMU) sensors are used in all aspects of human gait analysis, since they are small, robust, accurate, and affordable. Systems based on IMU sensors can be used to collect movement data in a more flexible and user-friendly manner, as they can be easily deployed and have longer battery life. They, also, provide rich information, as they allow for the accurate acquisition of 3D movement data, including the orientation of the tracked individual [35,36]. Moreover, while insoles are limited to being embedded in footwear, IMU sensors are not, and can be used in various configurations and numbers on an individual’s body [22,37], from smart devices and clothing items to fitness trackers [38] and medical devices [24]. Additionally, due to their non-obstructive characteristics, and long battery life, IMUs are suitable for long-term recording sessions. Thus, IMU-based sensing devices may be considered more suitable for the development of remote monitoring, continuous, home-based systems, with an improved user experience.

This work set out to evaluate the correlation of a specific set of gait features when extracted from two different systems, one based on pressure insoles, and one based on IMU sensors. The results of this comparison included both the statistical correlations between the extracted features from the two sources (insoles and IMUs), as well as an assessment of the importance of those features in building classification models for evaluating gait impairment. The results can be summarized in the following main findings. First, the study found a relatively high correlation between the common derived features from both systems (insoles and IMUs), indicating a small drop in accuracy when switching from pressure-insole-based devices to IMU sensor-based devices. However, it’s important to note that the features extracted from these two types of devices are neither exhaustive nor identical. Therefore, results may vary significantly when additional features are included. For instance, our analysis did not include double support and loading response features estimated from IMU sensor data, as we only considered features that could be estimated from a single IMU sensor. Second, the study conducted a feature importance analysis to assess the discriminating power of each individual feature. The analysis indicated that features from both sources (pressure insoles and IMU sensors) were important in training machine-learning classification models. Third, the study found that the performance of different machine learning models for gait impairment detection was similar, regardless of the feature set used. Fourth, using features from instrumented insoles, IMU-based devices placed on the shanks, or a combination of both, can lead to accurate detection of gait impairment with AUC values of 0.92 (Support Vector Machine), 0.89 (Gradient Boosting), and 0.93 (Support Vector Machine or Random Forest), respectively, when considering the highest scoring model. The models were constructed using only a subset of data generated during the “Walk Straight and Turn Test,” specifically those corresponding to the test iteration conducted with patients walking at a normal speed. This scenario is considered closer to real-world, practical ambulatory settings, which makes the developed models for gait impairment detection potentially more clinically relevant.

Finally, there are 2 points pertaining to the statistical analysis conducted for this work, and in particular, to the correlations, that should be discussed. First, for both, left/right side stance phases and single support, one can observe that the correlation resulting from the left foot data (r=0.94) is higher than the correlation from the right foot (r=0.84), with the latter still being excellent. This abnormality should be mainly attributed to the fact that the “Walk Straight and Turn Test” included in the analysis necessitates for the participants to perform a turn and not to, simply, walk in a straight line (i.e., compared to a treadmill test). Alternatively, it may have resulted due to specific unusual characteristics of the walking patterns of the participants. Second, it should be noted that in our study neither system could be considered as the ground truth, and the differences notwithstanding, it is clear that there is a very strong agreement between them, with the main kinematic features being highly correlated.

## 5. Conclusions

In conclusion, this work indicated that data from both instrumented insoles and IMU-based systems resulted in the extraction of highly correlated gait kinematic features. Moreover, data from both sources can be used to build accurate Machine Learning models, although models utilizing features from the data of the pressure insoles did have slightly better performance compared to those utilizing features only from the IMU-based system’s data. Nonetheless, the relative difference is small and it does not affect the capacity of the two systems to be used for gait assessment. In summary, IMU-based devices can be recommended for use by patients in home environments, for the evaluation of gait-related symptoms, without significant deviations in their accuracy, compared to systems based on pressure insoles. Concluding, although the results are promising, further studies are required in order to compare the feasibility, and the performance, of both aforementioned methods in ambulatory care, while patients perform their daily activities in their natural environment.

## Figures and Tables

**Table 1:** Demographic and medical information regarding the patients of the clinical study. Values are presented as Mean ± Standard Deviation. “DCIP” stands for “Dopamine Continuous Infusion Pumps”. “Lev. equiv. dose” stands for “Levodopa equivalent dose”. MDS-UPDRS Part III represents the total score of the 3rd part. The control subset score refers to the total score of items 3.9 to 3.14 of Part III of the MDS-UPDRS. Adapted with permission from [25]. 2022, Chatzaki et al.

Patient Information	OFF State	ON State	DCIP
Number of participants	17	17	2
Gender ratio (M/F)	14/3	14/3	0/2
Age span (years)	29–76	29–76	63–72
Average age (years)	62±11	62±11	68±5
Average height (cm)	172±9	172±9	162±7
Average weight (kg)	78±17	78±17	72±12
Disease duration (years)	10±11	10±11	17±6
Lev. equiv. dose (mg)	N/A	578±174	1147±671
MDS-UPDRS Part III	42±21	30±20	33±28
Control subset score	8±7	5±6	9±6

**Table 2:** The MDS-UPDRS 3.10 (Gait) Item expert rating distribution for the 19 patients that took part in the study. In the first column, all available rating options for the MDS-UPDRS Item 3.10 are listed. In the column corresponding to the OFF state rating distribution, there are only 17 patients rated in total. The reason being that 2 patients were using the DCIP, thus they could not be rated in the OFF state.

Item Rating	OFF State	ON State
0	1	4
1	10	11
2	4	2
3	0	2
4	2	0

**Table 3:** Timing of gait events identified from the data of the pressure insoles, along with the conditions for their identification. The column named “Time point” denotes the id of the time point that the respective event occurred. The time points are used for the calculation of gait features as they are described in Table 4.

Gait Event	Time Point	Insole Foot Placement	Identification Condition
Right heel strike	$t_{01}$	Right foot	(*Heel* > *Force Threshold Heel*) ∧ (*Toe* ≤ *Force Threshold Toe*)
Left toe off	$t_{02}$	Left foot	(*Heel* ≤ *Force Threshold Heel*) ∧ (*Toe* ≤ *Force Threshold Toe*)
Right toe strike	$t_{03}$	Right foot	(*Heel* > *Force Threshold Heel*) ∧ (*Toe* > *Force Threshold Toe*)
Right heel off	$t_{04}$	Right foot	(*Heel* ≤ *Force Threshold Heel*) ∧ (*Toe* > *Force Threshold Toe*)
Left heel strike	$t_{05}$	Left foot	(*Heel* > *Force Threshold Heel*) ∧ (*Toe* ≤ *Force Threshold Toe*)
Right toe off	$t_{06}$	Right foot	(*Heel* ≤ *Force Threshold Heel*) ∧ (*Toe* ≤ *Force Threshold Toe*)
Left toe strike	$t_{07}$	Left foot	(*Heel* > *Force Threshold Heel*) ∧ (*Toe* > *Force Threshold Toe*)
Left heel off	$t_{08}$	Left foot	(*Heel* ≤ *Force Threshold Heel*) ∧ (*Toe* > *Force Threshold Toe*)

**Table 4:** The gait features extracted from the data of the instrumented insoles based on the timing of the gait events [17]. Some of the gait features were also calculated as a percentage over the gait cycle. The time points are the ones listed in Table 3.

Gait Feature	Feature Calculation	Percentage Calculation
Right single support	$a_{01}=t_{02}^{\prime }-t_{05}$	$a_{15}=a_{01}/a_{09}$
Left single support	$a_{02}=t_{01}^{\prime }-t_{06}$	$a_{16}=a_{02}/a_{09}$
Double support	$a_{03}=a_{06}+a_{10}$	$a_{17}=a_{03}/a_{09}$
Right side stance phase	$a_{04}=t_{06}-t_{01}$	$a_{18}=a_{04}/a_{09}$
Left side stance phase	$a_{05}=t_{02}^{\prime }-t_{05}$	$a_{19}=a_{05}/a_{09}$
Right loading response	$a_{06}=t_{02}-t_{01}$	$a_{20}=a_{06}/a_{09}$
Right terminal stance	$a_{07}=t_{04}-t_{02}$	$a_{21}=a_{07}/a_{09}$
Right pre-swing	$a_{08}=t_{05}-t_{04}$	$a_{22}=a_{08}/a_{09}$
Right gait cycle duration	$a_{09}=t_{01}^{\prime }-t_{01}$	Not calculated
Left loading response	$a_{10}=t_{06}-t_{05}$	$a_{23}=a_{10}/a_{09}$
Left terminal stance	$a_{11}=t_{07}-t_{06}$	$a_{24}=a_{11}/a_{09}$
Left pre-swing	$a_{12}=t_{01}^{\prime }-t_{07}$	$a_{25}=a_{12}/a_{09}$
Left gait cycle duration	$a_{13}=t_{02}^{\prime }-t_{02}$	Not calculated
Walking cadence	$a_{14}=1/\left(t_{02}^{\prime }-t_{01}\right)$	Not calculated

**Table 5:** Correlation and intra-class correlation between the set of common features extracted from data of pressure insoles and IMU-based sensing devices.

Gait Feature	Pearson Correlation (r)	Intra-Class Correlation (ICC)
Walking cadence	0.98	0.97
Right gait cycle duration	0.97	0.96
Left gait cycle duration	0.98	0.98
Right single support	0.86	0.83
Left single support	0.94	0.91
Right side stance phase	0.88	0.91
Left side stance phase	0.93	0.92
Number of steps	0.94	0.91

**Table 6:** Classifiers were trained using the features extracted from the data of instrumented insoles, the features extracted from the data of the IMU-based devices, and a combination of features extracted from both aforementioned data sources. In the table, “SVM” stands for Support Vector Machine, “RF” for Random Forest, “GB” for Gradient Boosting, and “AB” for AdaBoost.

Metric	Insole					IMUs					Insoles & IMUs			
	SVM	RF	GB	AB		SVM	RF	GB	AB		SVM	RF	GB	AB
AUC	0.92	0.92	0.91	0.85		0.85	0.86	0.89	0.76		0.93	0.93	0.90	0.83
Accuracy	0.89	0.91	0.88	0.88		0.85	0.85	0.86	0.83		0.88	0.89	0.87	0.87
F1-Score	0.88	0.90	0.88	0.88		0.84	0.85	0.86	0.83		0.87	0.89	0.87	0.87
Precision	0.89	0.90	0.89	0.89		0.84	0.85	0.86	0.84		0.88	0.89	0.88	0.88
Recall	0.89	0.91	0.88	0.88		0.85	0.85	0.86	0.83		0.88	0.89	0.87	0.87

**Table 7:** This table presents 3 sets of features used as inputs to the described machine learning classification algorithms. The first set includes features extracted from the data of the insoles, the second includes features extracted from the data of the IMU-based system, and the third includes a subset of the combination of the previous two sets. In the third set the designation “ins.” refers to the insole-based system, while “IMU” to the IMU-based system. Those sets are presented in decreasing order of importance from top to bottom. The feature importance values were calculated using the Shapley Additive Explanations (SHAP) framework.

	Insole Features	IMU Features	Insole & IMU Features
F01	Right loading response (%, std)	Accel. max energy (asym., std)	Right load. response (%, std, ins.)
F02	Left loading response (%, mean)	Walking cadence (asym.)	Left pre-swing (mean, ins.)
F03	Pre-swing variability	Range of shank’s motion	Total accel. energy (asym., IMU)
F04	Left loading response (%, std)	Total gyroscope’s energy	Total accelerometer’s energy (IMU)
F05	Left pre-swing (mean)	Normalized stride length	Pre-swing variability (ins.)
F06	Right loading response (std)	Cumul. accelerometer’s energy	Normalized stride length (IMU)
F07	Left loading response (std)	Norm. walking speed (asym.)	Right load. response (std, ins.)
F08	Right terminal stance (%, mean)	Maximum rotation	Left load. response (std, ins.)
F09	Left single support (std)	Number of steps (count)	Range of shank’s motion (IMU)
F10	Right terminal stance (mean)	Contact time (asym.)	Norm. walk. speed (asym., IMU)

## Data Availability

The data used in this work, comprised of the IMU- and the insole-generated dataset, are available from the corresponding author upon reasonable request.

## Abbreviations

The following abbreviations are used in this manuscript:

TO	Toe Off
LF	Left Foot
RF	Right Foot
FC	Final Contact
IC	Initial Contact
MS	Maximum Swing
MD	Monitoring Device
PD	Parkinson’s Disease
TUG	Timed Up and Go Test
SVM	Support Vector Machine
ADL	Activities of Daily Living
IMU	Inertial Measurement Unit
AUC	Area Under (the ROC) Curve
SHAP	Shapley Additive Explanations
UPDRS	Unified Parkinson’s Disease Rating Scale
LSTM	Long Short-Term Memory Neural Network
MDS	International Parkinson and Movement Disorder Society
